# Partner number and use of COVID-19 risk reduction strategies during initial phases of the pandemic in British Columbia, Canada: a survey of sexual health service clients

**DOI:** 10.17269/s41997-021-00566-9

**Published:** 2021-11-03

**Authors:** Mark Gilbert, Hsiu-Ju Chang, Aidan Ablona, Travis Salway, Gina Ogilvie, Jason Wong, Laurence Campeau, Catherine Worthington, Daniel Grace, Troy Grennan

**Affiliations:** 1 Clinical Prevention Services, British Columbia Centre for Disease Control, 655 West 12th, Vancouver, British Columbia V5Z 4R4 Canada; 2 School of Population and Public Health, University of British Columbia, Vancouver, British Columbia Canada; 3 Faculty of Health Sciences, Simon Fraser University, Burnaby, British Columbia Canada; 4 Women’s Health Research Institute, BC Women’s, Vancouver, British Columbia Canada; 5 Vancouver Island Health Authority, Victoria, British Columbia Canada; 6 School of Public Health and Social Policy, University of Victoria, Victoria, British Columbia Canada; 7 Dalla Lana School of Public Health, University of Toronto, Toronto, Ontario Canada; 8 Division of Infectious Diseases, University of British Columbia, Vancouver, British Columbia Canada

**Keywords:** COVID-19, Sexual behaviour, Public health, Health promotion, COVID-19, comportement sexuel, santé publique, promotion de la santé

## Abstract

**Objectives:**

Initial public health guidance related to sex and COVID-19 infection focused on reducing partner number. We characterized individuals having a higher partner number during the initial phases of the pandemic.

**Methods:**

In British Columbia, the initial wave of COVID-19 cases was from March 14 to May 19, 2020, followed by gradual lifting of public health restrictions. We conducted an e-mail survey of existing sexual health service clients during the period of July 23 to August 4, 2020. We used bivariate logistic regression to examine the association between the reported number of sexual partners since the start of the pandemic and key variables (level of significance *p* < 0.01).

**Results:**

Of the 1196 clients in our final sample, 42% reported 2+ partners since the start of the pandemic, with higher odds among participants who were men who have sex with men, and single or in open relationships prior to the pandemic. This group was more likely to perceive stigma associated with having sex during the pandemic, and had the highest use of strategies to reduce risk of COVID-19 infection during sexual encounters (mainly focused on reducing/avoiding partners, such as masturbation, limiting sex to a “bubble”, and not having sex).

**Conclusion:**

Sexual health service clients in BC with 2+ partners during the initial phases of BC’s pandemic used strategies to reduce their risk of COVID-19 infection during sex. Our study provides support for a harm reduction approach to guidance on COVID-19 risk during sex, and highlights the need for further research on stigma related to having sex during the COVID-19 pandemic.

**Supplementary Information:**

The online version contains supplementary material available at 10.17269/s41997-021-00566-9.

## Résumé

**Objectifs:**

Les premières directives de santé publique sur les rapports sexuels et l’infection par la COVID-19 portaient sur la réduction du nombre de partenaires. Nous avons caractérisé les personnes ayant eu de nombreux partenaires au cours des phases initiales de la pandémie.

**Méthode:**

En Colombie-Britannique, la première vague de cas de COVID-19 a déferlé du 14 mars au 19 mai 2020, puis a été suivie d’une levée progressive des restrictions sanitaires. Nous avons administré un sondage par courriel auprès d’usagers existants des services de santé sexuelle entre le 23 juillet et le 4 août 2020. Nous avons procédé par régression logistique bivariée pour examiner l’association entre le nombre déclaré de partenaires sexuels depuis le début de la pandémie et certaines variables clés (seuil de signification *p* < 0,01).

**Résultats:**

Sur les 1196 usagers de notre échantillon final, 42 % ont dit avoir eu 2 partenaires ou plus depuis le début de la pandémie, avec une probabilité plus élevée chez les participants qui étaient des hommes ayant des relations sexuelles avec des hommes et ceux qui étaient célibataires ou dans une relation ouverte avant la pandémie. Ce groupe était plus susceptible de percevoir une stigmatisation des relations sexuelles durant la pandémie, et il a présenté l’utilisation la plus élevée de stratégies pour réduire le risque de contracter la COVID-19 lors de rapports sexuels (principalement en réduisant partiellement ou à zéro le nombre de partenaires, p. ex. en se masturbant, en limitant ses partenaires aux membres de sa « bulle» ou en s’abstenant de tout rapport sexuel).

**Conclusion:**

Les usagers des services de santé sexuelle de la Colombie-Britannique ayant eu 2 partenaires ou plus durant les premières phases de la pandémie dans la province ont employé des stratégies pour réduire leur risque de contracter la COVID-19 durant les rapports sexuels. Notre étude confirme la validité d’une approche de réduction des méfaits dans les directives sur le risque de contracter la COVID-19 durant les rapports sexuels et souligne le besoin de pousser la recherche sur la stigmatisation des rapports sexuels durant la pandémie de COVID-19.

## Introduction

Societal measures to respond to the COVID-19 pandemic led to disruptions across multiple aspects of people’s lives, including expressions of their sexuality (Döring, 2020). Stringent contact restrictions and lockdowns may have limited opportunities for sex, such that “those living alone…are effectively mandated to a period of celibacy to confine the pandemic” (Lehmiller et al., 2020). While there is no direct evidence of sexual transmission of SARS-CoV-2, infection may be acquired during sexual encounters through exposure to sex partners with infection.

Accordingly, many agencies released recommendations to reduce the risk of COVID-19 transmission through sex, with early messaging focused on reducing sexual partner number. For example, in British Columbia (BC), Canada, the first public health guidance on sex and COVID-19 (dated April 29, 2020) recommended the safest sex options being masturbation and avoiding sex with anyone outside the household (BC Centre for Disease Control, 2020a). However, there may be unintended consequences of this early messaging, including “prescriptive narratives” on social and news media to encourage or force people to stop having casual sex, possibly leading to stigma, shame, and moral panic about having sex during the COVID-19 pandemic (DiManno, 2020; Döring, 2020; Kibbe, 2020; Logie & Turan, 2020; Rodriguez, 2020).

Research conducted between March and May 2020 demonstrated the initial impacts of the global pandemic on sexuality and sexual behaviours, including measures congruent with public health recommendations such as increases in virtual sex and reductions in number of partners, to varying degrees (Alpalhao & Filipe, 2020; de Sousa et al., 2020; Döring, 2020; Grace et al., 2021; Hammoud et al., 2020; Li et al., 2020; McKay et al., 2020; Mestre-Bach et al., 2020; Sanchez et al., 2020). However, the extent to which these sexual behaviour changes persisted beyond March–May 2020 was less well described. This was particularly relevant as in many countries, COVID-19 case counts had decreased, societal restrictions were progressively lifted, and concern about COVID-19 may have eased, leading to increased population mixing—and potentially, increases in numbers of sexual partners (Rodriguez, 2020). Also, around this time, many jurisdictions (including BC (BC Centre for Disease Control, 2020b)) shifted to a more sex-positive and harm reduction approach to guidance, recognizing the importance of sex for well-being, and inclusive of measures to reduce risk of COVID-19 infection during casual or transactional sex, often following the lead of community-based organizations (ACT, 2020). However, the extent to which risk reduction measures to reduce COVID-19 risk have been taken up had not yet been well described.

**Fig. 1:**
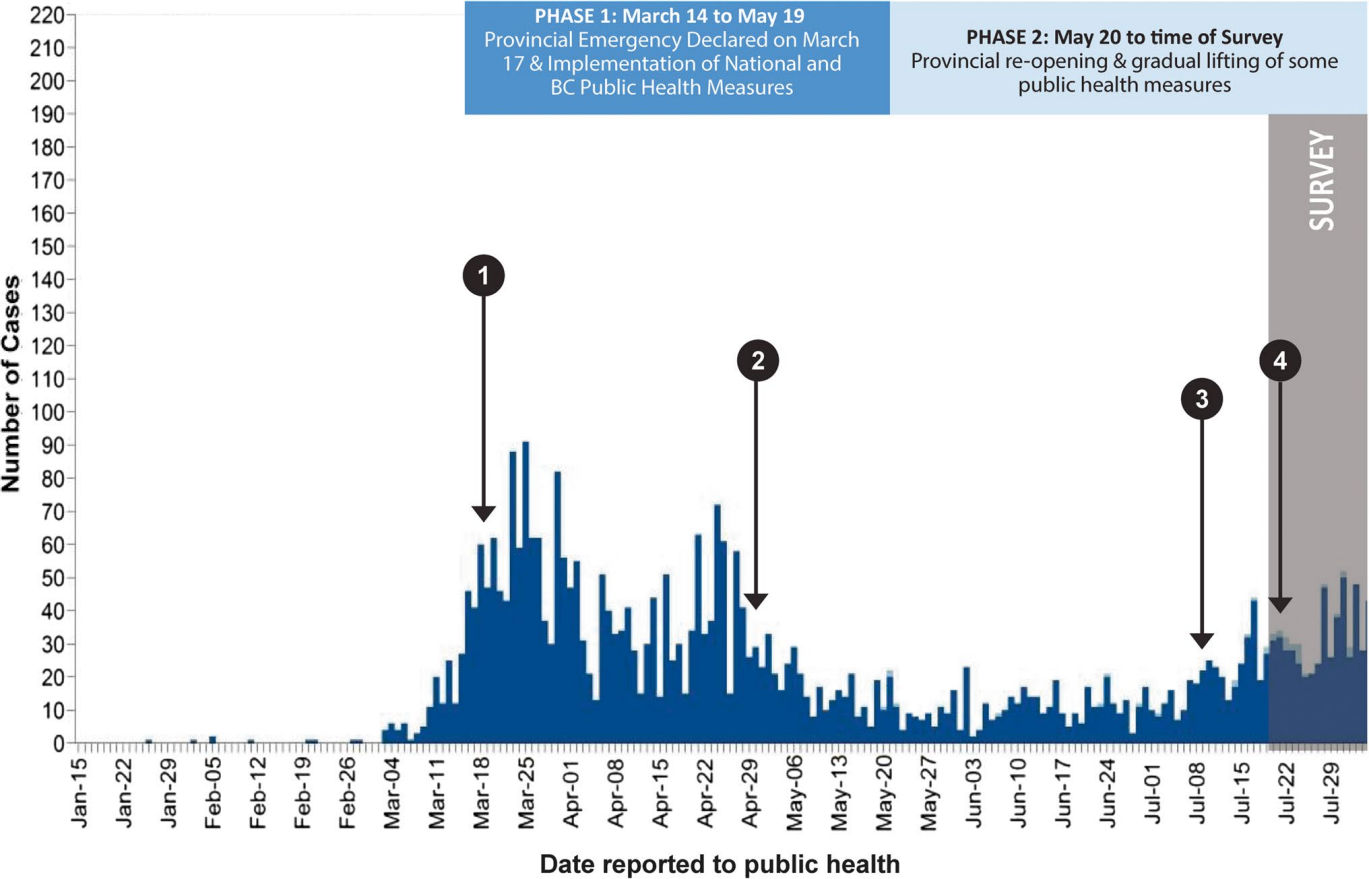
Reported COVID-19 cases in British Columbia, initial phases of the pandemic, and relevant key events prior to the survey. Legend: 1: March 18, 2020, BCCDC STI clinic closures and service restrictions. 2: April 29, First BC guidance on sex and COVID-19 released, focused on restricting sex to self or household partners. 3: July 9, Second more sex-positive BC guidance on sex and COVID-19 released, focused on harm reduction. 4: July 22–24, Media attention to revised BC guidance on sex and COVID-19

The primary objective of our study was to identify characteristics associated with having higher partner number, and understand how partner number changed during the initial phases of the COVID-19 pandemic in BC (between mid-March and August 2020) among existing sexual health service clients. Our secondary objectives were to measure the extent to which this population was worried about acquiring COVID-19 infection during sexual encounters, and the uptake of promoted strategies to reduce COVID-19 risk during encounters. We hypothesized most individuals would report taking measures to reduce their risk of exposure to COVID-19 through sex, including reduced number of partners. We also hypothesized that since—at the time of our survey—there were lower numbers of COVID-19 cases in BC and relaxing of public health measures (Fig. 1), participants would have less worry about COVID-19 and might be relaxing risk reduction measures initially taken.

## Methods

We used the CHERRIES checklist for reporting results of Internet surveys to inform our description of study methods (see Supplemental Content) (Eysenbach, 2004). Our study design and interpretation of results were informed by principles of harm reduction (e.g. focus on reducing not eliminating harms, affirming individual responsibility, and avoidance of perpetuating stigma).

### Design, setting

We conducted a cross-sectional e-mail survey between July 21 and August 4, 2020, during a period (phase 2) of provincial re-opening and gradual lifting of public health measures following the initial wave of COVID-19 cases (phase 1, Fig. 1). Using previously established methods (Gilbert et al., 2018), we recruited participants from existing clients of a provincial sexually transmitted infection (STI) clinic and GetCheckedOnline (GCO), BC’s Internet-based STI testing service, both operated by the BC Centre for Disease Control (BCCDC). The STI clinic, located in Vancouver, BC, saw over 10,000 clients per year prior to the pandemic. GCO is available in eight communities across BC, with 66% of clients residing in the greater Vancouver area and over 11,000 tests conducted in 2019. We have previously demonstrated overlap between the clients of these services (Gilbert et al., 2018).

### Survey development

Survey items were adapted from the literature and our prior research (Gilbert et al., 2018; Sanchez et al., 2020), or developed de novo with input from sexual health service providers at the BCCDC and our research team’s Community Advisory Board (CAB). The survey was pilot tested with eight members of the CAB and revised accordingly. The final online survey contained 33 items in total (one per page), and used adaptive questioning to minimize the number of items for completion by each. Participants had the ability to go backwards and forwards in the survey to review or edit answers, and could save answers and continue the survey at a later date. The survey was available in English only.

The survey included questions on sexual partners, worry about COVID-19 risk, use of COVID-19 risk reduction strategies included in the revised BC guidance (BC Centre for Disease Control, 2020b), perceived stigma, and socio-demographics. We asked questions related to changes in partner number in phase 1 (defined as “March to mid-May 2020”) relative to the pre-pandemic period, and phase 2 (defined as “mid-May to time of survey”) relative to phase 1 (for survey instrument, see Supplemental Content). Four response options were provided: decreased, no change, increased, and not applicable.

### Recruitment and data collection

We identified existing clients at least 16 years old who had visited the BCCDC STI clinic or tested using GCO in the year prior to the pandemic (March 15, 2019–March 17, 2020) and who had previously consented to be contacted for research and provided an e-mail address. The e-mail invitation sent to these clients contained a generic recruitment message with a link to the online survey. Two distinct URLs were created, one for STI clinic clients and one for GCO clients, to allow for comparison between these two samples. The survey landing page included necessary information for informed consent, indicating participation was voluntary and including additional eligibility criteria (ability to complete surveys in English, and not having completed the survey previously). Proceeding to the survey was taken as consenting to participate. Participants were offered an opportunity to enter into a draw for a $200 CAD gift card. The initial recruitment email was sent on July 21, 2020, with follow-up reminders on July 23, 25, and 29; the survey was closed on August 4, 2020. Data were collected using REDCap, with no personal identifiers collected. Survey completion was tracked based on participants clicking on “submit” at the end of the survey. After the survey closed, survey data were downloaded and stored in a secure data repository.

### Analysis

As attrition was high in the beginning sections of the survey, only completed questionnaires were included in the analysis. Our primary outcome of interest was number of sexual partners reported since the start of the pandemic, which we categorized as 0–1 partner or 2+ partners. We also looked at change in the number of sexual partners during the pandemic prior to the survey. Based on our prior hypotheses and review of dominant sexual behaviour patterns observed in the data, we classified partner number into four groups labeled according to predominant patterns as follows: no change in both phases; decreased in both phases; increased starting in phase 1; increased in phase 2 (for full details of the classification method, see Figure, Supplemental Content). We applied the same classification to other similarly structured variables (e.g. changes in opportunities to have sex). We created a composite variable for perceived stigma, based on the level of agreement with two questions: “I would feel ashamed if people know about my sex life during the COVID-19 pandemic” and “Other people will judge me for having sex during the COVID-19 pandemic”.

We conducted bivariate comparisons of key behavioural and socio-demographic variables and number of partners using Chi-square, Fisher’s exact, or *t*-test as appropriate. Due to multiple comparisons, we conservatively set the level of significance at *p* < 0.01. We used logistic regression to calculate odds ratios (ORs) and 99% confidence intervals. All analyses were conducted using R V.3.52.

### Ethics approval

Ethics approval for this study was granted by the University of British Columbia’s Behavioural Research Ethics Board (certificate #H18-00,437).

## Results

### Recruitment outcomes

A total of 4212 eligible clients were invited, 1518 (36%) of whom participated, with 79% (1198/1518) completing the survey. The response rate among GCO clients (2618 eligible, 42% (1087/2618) participating, and 78% (851/1087) completed) was higher than that for the STI clinic clients (1594 eligible, 27% (431/1594) participating, and 81% (347/431) completed). Of the 1198 participants completing the survey, two did not answer the question related to partner number, resulting in 1196 participants as the final sample. Overall, the median age was 32 years, with 48% identifying as a man, 47% as a woman, and 5% as non-binary, gender-fluid, or other gender (Table 1). In total, 35% of the participants were women who have sex with men only, 25% were men who have sex with men, 22% were men who have sex with women only, and 11% were women who have sex with women. The majority of participants were white (72%), with 4% identifying as Indigenous and 24% as another racialized minority. Most participants (89%) had a greater than high school education.

**Table 1:** Socio-demographics, partner types, and sexual behaviours of sexual health service clients stratified by number of partners since start of the pandemic in BC

Variable	Total *N* = 1196	0–1 partner *N* = 690	2+ partners *N* = 506	*p*-value	Unadjusted odds ratios [99% CI]
Age, years: median [inter-quartile range]	32 [27–41]	32 [26–40]	34 [28–41]	0.019	1.01 [1.00, 1.03]
Gender identity				** < 0.001**	
Man	577/1194 (48%)	274/690 (40%)	303/504 (60%)		**2.26 [1.65, 3.10]**
Woman	563/1194 (47%)	378/690 (55%)	185/504 (37%)		Reference
Non-binary / gender-fluid / other	54/1194 (5%)	38/690 (6%)	16/504 (3%)		0.86 [0.37, 1.86]
Sexual identity				** < 0.001**	
Straight (heterosexual)	625/1190 (53%)	423/685 (62%)	202/505 (40%)		Reference
Sexual minority (e.g. gay, lesbian, homosexual, queer, pansexual)	565/1190 (47%)	262/685 (38%)	303/505 (60%)		**2.42 [1.78, 3.31]**
Gender of sex partners prior to the pandemic				** < 0.001**	
Men who have sex with women only	258/1167 (22%)	161/664 (24%)	97/503 (19%)		Reference
Men who have sex with men	291/1167 (25%)	94/664 (14%)	197/503 (39%)		**3.48 [2.20, 5.56]**
Women who have sex with men only	414/1167 (35%)	291/664 (44%)	123/503 (24%)		0.70 [0.46, 1.08]
Women who have sex with women	124/1167 (11%)	67/664 (10%)	57/503 (11%)		1.41 [0.80, 2.50]
Other	80/1167 (7%)	51/664 (8%)	29/503 (6%)		0.94 [0.47, 1.86]
Race/ethnicity				0.489	
Indigenous	46/1183 (4%)	25/682 (4%)	21/501 (4%)		1.11 [0.50, 2.42]
Racialized minority, non-Indigenous	289/1183 (24%)	175/682 (26%)	114/501 (23%)		0.86 [0.60, 1.22]
White	848/1183 (72%)	482/682 (71%)	366/501 (73%)		Reference
Greater than high school education (vs. less)	1057/1189 (89%)	617/684 (90%)	440/505 (87%)	0.115	0.74 [0.46, 1.19]
Relationship at start of COVID-19 pandemic				** < 0.001**	
Single	466/1190 (39%)	232/686 (34%)	234/504 (46%)		**7.60 [4.73, 12.66]**
In a monogamous relationship	350/1190 (29%)	309/686 (45%)	41/504 (8%)		Reference
In an open relationship	323/1190 (27%)	123/686 (18%)	200/504 (40%)		**12.25 [7.40, 21.00]**
Other	51/1190 (4%)	22/686 (3%)	29/504 (6%)		**9.93 [4.31, 23.60]**
Description of sex partners					
Person(s) have sex with regularly (vs. not)	832/1043 (80%)	454/537 (85%)	378/506 (75%)	** < 0.001**	**0.54 [0.36, 0.81]**
New sexual partner (vs. not)	276/1043 (26%)	65/537 (12%)	211/506 (42%)	** < 0.001**	**5.19 [3.47, 7.93]**
Casual partner/hook-up (vs. not)	321/1043 (31%)	51/537 (9%)	270/506 (53%)	** < 0.001**	**10.90 [7.09, 17.25]**
Lives with partner(s) (vs. not)	322/1036 (31%)	186/536 (35%)	136/500 (27%)	0.011	0.70 [0.50, 1.00]
Change in partner number					
No change both phases	473/1135 (42%)	353/633 (56%)	120/502 (24%)	** < 0.001**	**0.58 [0.39, 0.86]**
Decreased in both phases	366/1135 (32%)	231/633 (36%)	135/502 (27%)		Reference
Increased in phase 1	57/1135 (5%)	9/633 (1%)	48/502 (10%)		**9.13 [3.71, 27.13]**
Increased in phase 2	239/1135 (21%)	40/633 (6%)	199/502 (40%)		**8.51 [5.11, 14.68]**
Change in interest in sex					
No change both phases	415/1186 (35%)	241/684 (35%)	174/502 (35%)	**0.001**	1.48 [0.93, 2.39]
Decreased in both phases	189/1186 (16%)	127/684 (19%)	62/502 (12%)		Reference
Increased in phase 1	255/1186 (22%)	154/684 (23%)	101/502 (20%)		1.34 [0.80, 2.27]
Increased in phase 2	327/1186 (28%)	162/684 (24%)	165/502 (33%)		**2.09 [1.28, 3.43]**
Change in opportunities to have sex					
No change both phases	226/1162 (19%)	161/664 (24%)	65/498 (13%)	** < 0.001**	0.70 [0.44, 1.10]
Decreased in both phases	414/1162 (36%)	262/664 (39%)	152/498 (31%)		Reference
Increased in phase 1	133/1162 (11%)	97/664 (15%)	36/498 (7%)		0.64 [0.36, 1.11]
Increased in phase 2	389/1162 (33%)	144/664 (22%)	245/498 (49%)		**2.93 [2.02, 4.29]**
Change in use of dating/hook-up apps to meet other people in person					
No change both phases	243/852 (29%)	144/414 (35%)	99/438 (23%)	** < 0.001**	0.91 [0.58, 1.41]
Decreased in both phases	336/852 (39%)	191/414 (46%)	145/438 (33%)		Reference
Increased in phase 1	96/852 (11%)	32/414 (8%)	64/438 (15%)		**2.63 [1.43, 5.01]**
Increased in phase 2	177/852 (21%)	47/414 (11%)	130/438 (30%)		**3.64 [2.18, 6.22]**
Feel ashamed if people knew about my sex life / others will judge me for having sex during the pandemic				** < 0.001**	
Strongly agree / agree	461/1190 (39%)	192/687 (28%)	269/503 (53%)		**2.96 [2.16, 4.08]**
Neither agree nor disagree / disagree / strongly disagree	729/1190 (61%)	495/687 (72%)	234/503 (47%)		Reference

BOLD: 95% confidence interval excludes 1

### Overall results

The greatest proportion (58%) of participants had 0–1 partner since the start of the pandemic (i.e. both phases), with 42% having 2+ partners (Table 1). More participants indicated no change in partner numbers in both phases of the pandemic (42%) or a decrease in both phases (32%); the remaining participants reported an increase in partner number, with 5% during phase 1 and 21% in phase 2 (see Supplemental Content). Most participants described their sex partners as persons they had sex with regularly (80%) and 31% lived with their sex partner(s).

Over a third (39%) of participants agreed that they would feel ashamed if people knew about their sex life and/or others would judge them for having sex during the pandemic (perceived stigma). Overall, 64% of participants were somewhat (38%), very (14%), or extremely worried (12%) about getting COVID-19 during sexual encounters in phase 1 of the BC pandemic (Table 2). Most participants (65%) reported no change in their level of worry during phase 2, with 26% being less worried and 9% being more worried.

**Table 2:** Worry about COVID-19 risk during sex and use of risk reduction strategies among sexual health service clients stratified by number of partners since start of the pandemic in BC

Variable	Total *N* = 1196	0–1 partner *N* = 690	2+ partners *N* = 506	*p* value	Unadjusted odds ratios [99% CI]
Worry about getting COVID-19 during sexual encounters during phase 1				** < 0.001**	
Not at all worried	421/1189 (35%)	311/683 (46%)	110/506 (22%)		Reference
Somewhat worried	454/1189 (38%)	209/683 (31%)	245/506 (48%)		**3.31 [2.29, 4.84]**
Very worried	169/1189 (14%)	81/683 (12%)	88/506 (17%)		**3.07 [1.89, 5.03]**
Extremely worried	145/1189 (12%)	82/683 (12%)	63/506 (12%)		**2.17 [1.29, 3.65]**
Worry about getting COVID-19 during sexual encounters during phase 2				** < 0.001**	
Less worried	307/1187 (26%)	118/682 (17%)	189/505 (37%)		**3.20 [2.24, 4.60]**
No change	770/1187 (65%)	513/682 (75%)	257/505 (51%)		Reference
More worried	110/1187 (9%)	51/682 (7%)	59/505 (12%)		**2.31 [1.36, 3.94]**
Use of strategies to reduce risk of getting or passing COVID-19 infection during sexual encounters (any) (vs. not)	1086/1194 (91%)	605/689 (88%)	481/505 (95%)	** < 0.001**	**2.78 [1.54, 5.34]**
Risk reduction strategies used* (vs. not)					
Masturbation	582/1086 (54%)	293/605 (48%)	289/481 (60%)	** < 0.001**	**1.60 [1.17, 2.21]**
Asking partners about symptoms of COVID-19 or their COVID-19 precautions	527/1086 (49%)	211/605 (35%)	316/481 (66%)	** < 0.001**	**3.58 [2.58, 4.99]**
Limiting sex to a small number of regular partners (“bubble”)	475/1086 (44%)	150/605 (25%)	325/481 (68%)	** < 0.001**	**6.32 [4.48, 9.00]**
Not having sex	480/1086 (44%)	282/605 (47%)	198/481 (41%)	0.083	0.80 [0.58, 1.10]
Avoiding sex if feeling unwell or have symptoms of COVID-19	452/1086 (42%)	168/605 (28%)	284/481 (59%)	** < 0.001**	**3.75 [2.69, 5.25]**
Limiting partner number (e.g. reducing number of casual partners, avoiding group sex)	448/1086 (41%)	144/605 (24%)	304/481 (63%)	** < 0.001**	**5.50 [3.90, 7.80]**
Limiting sex to a person/people you live with	312/1086 (29%)	198/605 (33%)	114/481 (24%)	**0.001**	**0.64 [0.45, 0.91]**
Having online or virtual sex	173/1086 (16%)	83/605 (14%)	90/481 (19%)	0.032	1.45 [0.94, 2.23]
Washing hands with soap and water before and after sex	370/1086 (34%)	133/605 (22%)	237/481 (49%)	** < 0.001**	**3.45 [2.45, 4.88]**
Precautions during sex (e.g. avoiding kissing or saliva contact/exchange; avoiding rimming; wearing a face mask during sex; avoiding face-to-face contact; washing shared sex toys)	300/1086 (28%)	103/605 (17%)	197/481 (41%)	** < 0.001**	**3.38 [2.35, 4.90]**
Other	35/1086 (3%)	26/605 (4%)	9/481 (2%)	0.038	0.42 [0.14, 1.10]
Looked for, or received information about COVID-19 infection during sexual encounters (any)	760/1189 (64%)	396/686 (58%)	364/503 (72%)	** < 0.001**	**1.92 [1.39, 2.66]**
Sources of information:					
From a public health agency website (e.g. BCCDC website)	446/760 (59%)	224/396 (57%)	222/364 (61%)	0.245	1.20 [0.82, 1.76]
By searching online	422/760 (56%)	212/396 (54%)	210/364 (58%)	0.281	1.18 [0.81, 1.73]
Through social media	363/760 (48%)	198/396 (50%)	165/364 (45%)	0.224	0.83 [0.57, 1.21]
Through news media	333/760 (44%)	167/396 (42%)	166/364 (46%)	0.379	1.15 [0.79, 1.68]
Through friends, family, or a relationship or sex partner	292/760 (38%)	146/396 (37%)	146/364 (40%)	0.399	1.15 [0.78, 1.69]
From a community-based organization or health care provider	126/760 (17%)	55/396 (14%)	71/364 (20%)	0.047	1.50 [0.91, 2.51]
Other	21/760 (3%)	13/396 (3%)	8/364 (2%)	0.490	0.66 [0.19, 2.09]

*Excluding participants who did not report using any risk reduction strategy

BOLD: 95% confidence interval excludes 1

Almost all participants (91%) used strategies to reduce their risk of COVID-19 during sex (Table 2). The most commonly used strategies were related to avoiding or limiting partners, such as masturbation (54%), asking partners about symptoms of COVID-19 or their COVID-19 precautions (49%), limiting sex to a small number of regular partners (“bubble”; 44%), and not having sex (44%). In-person risk reduction strategies during sexual encounters were less common in the overall sample at 28% (e.g. avoiding kissing or face-to-face contact). Notably less common were strategies that were initial mainstays of public health communications about reducing risk of COVID-19 infection during sex, such as restricting sex to a person lived with (29% of sample). A total of 64% (762/1191) of participants had looked for or received information about COVID-19 risk and sex. The most common information sources included public health agency websites (59%), searching online (56%), and social media (48%). Just over a third (38%) had looked for or received information from friends, family, a relationship or sex partner.

### Characteristics associated with having 2+ partners

The odds of having 2+ partners were higher among men (unadjusted OR 2.26, 99% confidence interval [1.65, 3.10]), sexual minority participants (OR 2.42 [1.78, 3.31]), and men who have sex with men (OR 3.48, [2.20, 5.56]; Table 1). Age, race/ethnicity, and education were not associated with partner number.

Compared with being in a monogamous relationship at the start of the pandemic, all other relationship types had higher odds of having 2+ partners (single, OR 7.60 [4.73, 12.66]; open relationship, OR 12.25 [7.40, 21.00]; other relationship, OR 9.93 [4.31, 23.60]). The odds of having 2+ partners were lower among participants reporting regular sex partners (OR 0.54 [0.36, 0.81]) and higher for participants reporting new sex partners (OR 5.19 [3.47, 7.93]) and casual partners (OR 10.90 [7.09, 17.25]). A lower proportion of participants with 2+ partners lived with their partner(s), which bordered on significance (OR 0.70 [0.50, 1.00]). Participants reporting no change in partner number during the initial phases of the pandemic had lower odds of having 2+ partners (OR 0.58 [0.39, 0.86]), whereas participants reporting increases in partner number in phase 1 (OR 9.13 [3.71, 27.13]) and in phase 2 (OR 8.51 [5.11, 14.68]) had higher odds. During phase 2, the odds of having 2+ partners were higher among participants reporting increased interest in sex (OR 2.09 [1.28, 3.43]), increased opportunities to have sex (OR 2.93 [2.02, 4.29]), and increased use of dating/hook-up apps to meet other people in person (OR 3.64 [2.18, 6.22]). Respondents perceiving increased stigma were more likely to have 2+ partners (OR 2.96 [2.16, 4.08]).

Participants reporting any level of worry other than “not at all worried” about getting COVID-19 during sexual encounters during phase 1 had higher odds of having 2+ partners, as did participants reporting being less worried (OR 3.20 [2.24, 4.60]) and more worried in phase 2 (2.31 [1.36, 3.94]; Table 2). Participants reporting use of any strategy to reduce their risk of getting or passing COVID-19 infection during sexual encounters had significantly higher odds of having 2+ partners (2.78 [1.54, 5.34]). The same pattern (higher odds) was observed for each risk reduction strategy reported used, with the exception of not having sex or having online or virtual sex (not significantly associated with partner number), and lower odds among those limiting sex to a person/people they lived with (OR 0.64 [0.45, 0.91]). Looking for or receiving information about the risk of being exposed to COVID-19 infection during sexual encounters was positively associated with having 2+ partners (OR 1.92 [1.39, 2.66]).

## Discussion

In our sample of existing sexual health service clients, 58% reported having 0 or 1 partner since the start of the COVID-19 pandemic, and the odds of being in this group were higher for people reporting sex with regular partners and limiting sex to a person they lived with as a risk reduction strategy. In this group, 92% also reported no change or a decrease in partner number during both initial phases of BC’s pandemic. This suggests that a majority of participants in our survey both were at low risk for COVID-19 infection during sexual encounters and may have been following initial public health messaging on COVID-19 risk reduction during sex. Of the participants, 42% reported 2+ partners since the start of the pandemic and thereby were potentially at greater risk of exposure to COVID-19 infection during sexual encounters. The odds of having 2+ partners were higher among participants identifying as men or classified as men who have sex with men, who were single or in an open relationship prior to the pandemic, and who reported new or casual sex partners during the pandemic. Initial public health messaging was likely less relevant for this group, who were also more likely to perceive stigma related to having sex during the COVID-19 pandemic. Overall, our findings support concerns that early public health messaging related to COVID infection prioritized heterosexual sex and may have led to stigmatization of non-monogamous relationships (Kibbe, 2020; Logie & Turan, 2020; Rodriguez, 2020).

Encouragingly, over 90% of all participants reported using at least one strategy to reduce their risk of COVID-19 infection during sex, with use generally greatest among the 2+ partner group (including limiting partner number), as well as seeking out COVID risk reduction information. These findings confirmed our hypothesis that most individuals had reduced partner numbers and/or taken other measures to reduce risk of COVID-19 as a result of the pandemic, and suggest almost all were taking responsibility for protecting themselves and their partners from COVID-19 infection during the pandemic. Our findings also support our hypothesis that some participants did have less worry about COVID-19 and were relaxing risk reduction measures following the initial wave of COVID-19 cases in BC (phase 1). Participants with higher odds of having 2+ partners reported increases in partner number in phase 2, as well as increased interest in sex, opportunities to have sex, use of dating/hook-up apps, and less worry about COVID infection during this period.

The changes in partner number reported by participants during the first phase of BC’s pandemic are similar to findings from other studies (Alpalhao & Filipe, 2020; de Sousa et al., 2020; Döring, 2020; Grace et al., 2021; Hammoud et al., 2020; Li et al., 2020; McKay et al., 2020; Mestre-Bach et al., 2020; Sanchez et al., 2020). Our study adds to the literature on the impact of the COVID-19 pandemic on partner number and sexual behaviours over different phases of the pandemic response. Among men who have sex with men accessing a sexual health service in Melbourne, Australia, the mean number of partners decreased during lockdown and began to increase post-lockdown (with no change observed for women or men who have sex with women) (Chow et al., 2020). Similarly, our findings found varying patterns in partner number change among sexual health service clients, with an increase for some during the easing of public health restrictions in BC. However, for the majority, there was either no change or a decrease in partners during the two phases of BC’s pandemic prior to the survey, regardless of case counts or easing of public health restrictions.

While changes in sex practices in response to COVID-19 risk have been reported elsewhere (Coombe et al., 2020; de Sousa et al., 2020), we build on this by demonstrating that most individuals were concerned about their risk of acquiring COVID-19 infection during sex, had received or sought information about this risk, and deliberately used strategies to reduce their risk. Our study explored perceptions of stigma related to having sex during the COVID-19 pandemic leading to shame or concerns about judgement, which was most common among participants reporting 2+ partners. This may be related to public health restrictions in general (e.g. physical distancing) or could be an unintended outcome of public health or media messaging about avoiding having sex or creating the impression of sex being dangerous (Logie & Turan, 2020; Turban et al., 2020). Ours is an exploratory analysis only and further research on the nature of stigma related to having sex during the pandemic is welcomed, given its potential role as a barrier to accessing sexual health services and needed care during the pandemic, as we have found in prior research on STI testing and access to HIV prevention services (Gilbert et al., 2018; Grace et al., 2017). Taken together, our findings emphasize the importance of developing stigma mitigation strategies, including continued promotion of COVID-19 risk reduction strategies during sex that use approaches that are sex-positive, non-judgemental, and based on principles of harm reduction. These may need to be tailored to different subpopulations (e.g. messages for men who have sex with men), and evolve with changes to pandemic measures (e.g. vaccination status). Further research may be helpful in this regard.

We sampled existing sexual health service clients to reflect a population that is sexually active, which is a strength of our survey as changes in sexual behaviours due to COVID-19 may be more likely to be observed in this population. However, we recognize our results have limited generalizability to the sexual behaviours of populations that have not engaged in sexual health care; furthermore, our sample was predominantly white and well educated. Our results may also not be generalizable to other jurisdictions where COVID-19 case trends, public health restrictions, guidelines, media attention, and community norms differ from those of BC (Fig. 1). Other limitations of our paper included volunteer and social desirability bias which may result in underestimating the proportion having 2 or more partners, as individuals following public health guidelines may be likely to respond to the survey, or participants may be more likely to report reduced sex partners during the COVID-19 pandemic. Media attention to BC’s revised guidance on sex and COVID-19 during the survey period may also have introduced bias (CBC News, 2020). We asked participants about their own perception of relative changes in partner number over time and were unable to quantify these changes or compare with a pre-pandemic baseline number of partners. We also are not able to describe how use of risk reduction strategies may have changed during the two phases of our survey.

## Conclusion

The findings of our study demonstrated that while just under half of existing sexual health service clients reported 2+ partners (many reporting increases in partner number during phase 2 of BC’s initial pandemic), the uptake of strategies to reduce the risk of COVID-19 was high. Since the time of this survey in BC, there have been increases in COVID-19 cases and re-introduction of public health restrictions. Given concerns regarding growing public fatigue at public health COVID-19-related restrictions (Bricker, 2020), ongoing assessments of the impact of the pandemic on partner number and other sexual metrics over time are needed. It is also critical to further explore how the pandemic may be affecting access to sexual health services during the COVID-19 pandemic, and how this may be influenced by perceived and enacted stigma. Our findings do affirm calls to develop stigma reduction strategies which are informed by the lived experiences of COVID-19 patients and other intersecting stigmas (e.g. racism, homophobia) (Hart et al., 2021; Logie & Turan, 2020). We also recognize that the prolongation of measures which are good from a public health perspective for reducing COVID-19 transmission during sex may have negative, unintended long-term impacts considering the centrality of sexuality to lifelong health and well-being—a topic worthy of further research when we move past the pandemic crisis (Alpalhao & Filipe, 2020; Lehmiller et al., 2020).

## Contributions to knowledge

What does the study add to existing knowledge?

- Of the 506 (42%) existing sexual health service clients surveyed reporting 2+ partners during the pandemic, almost all (95%) were using strategies to reduce their risk of COVID-19 infection during sex (e.g. limiting sex to a “bubble”).
- Following the first wave of COVID-19 cases and easing of public health restrictions, participants reporting 2+ partners were more likely to report less worry about COVID-19, increases in partner number, opportunities to have sex, and use of dating apps.
- Overall, 39% of clients surveyed perceived stigma related to having sex during the COVID-19 pandemic (53% among participants with 2+ partners).

What are the key implications for public health interventions, practice, and policy?

- Our study provides support for concerns that initial public health messaging during the pandemic focused on reducing partner number (e.g. limit sex to people lived with) may have reinforced heterosexual norms and stigmatized non-monogamous relationships.
- Public health messaging for reducing risk of COVID-19 infection during sex which adopts harm reduction principles is recommended, including consideration of targeting to groups more likely to report 2+ partners during the pandemic (e.g. men who have sex with men).
- Based on this initial exploratory analysis, further research is needed to better characterize the nature of stigma related to having sex during the COVID-19 pandemic.

## Supplementary Information

Below is the link to the electronic supplementary material.

Supplementary file1 (PDF 456 KB)

## Data Availability

Data may be available upon request due to privacy/ethical restrictions.

## Code availability

R code for analysis may be available upon request.
