# Supplementary material for: Partner number and use of COVID-19 risk reduction strategies during initial phases of the pandemic in British Columbia, Canada: a survey of sexual health service clients
Source: Can J Public Health. 2021 Nov 3;112(6):973–83. doi: 10.17269/s41997-021-00566-9 (PMC8565172; doi:10.17269/s41997-021-00566-9)
Supplement: Supplementary file 1 — Supplementary file1 (PDF 456 KB) [file 41997_2021_566_MOESM1_ESM.pdf]

Mark Gilbert, et al. Changes in partner number and use of COVID-19 risk reduction strategies during initial phases of the pandemic in British Columbia, Canada: a survey of sexual health service clients. *Canadian Journal of Public Health.* (Corresponding author: Mark Gilbert, BC Centre for Disease Control. Mark.gilbert@bccdc.ca.)

Supplemental Content:

Figure: Classification of participants into four partner change groups based on relative changes in the number of sexual partners reported in phase 1 (March to mid-May, 2020) and Phase 2 (mid-May to time of July/August 2020)

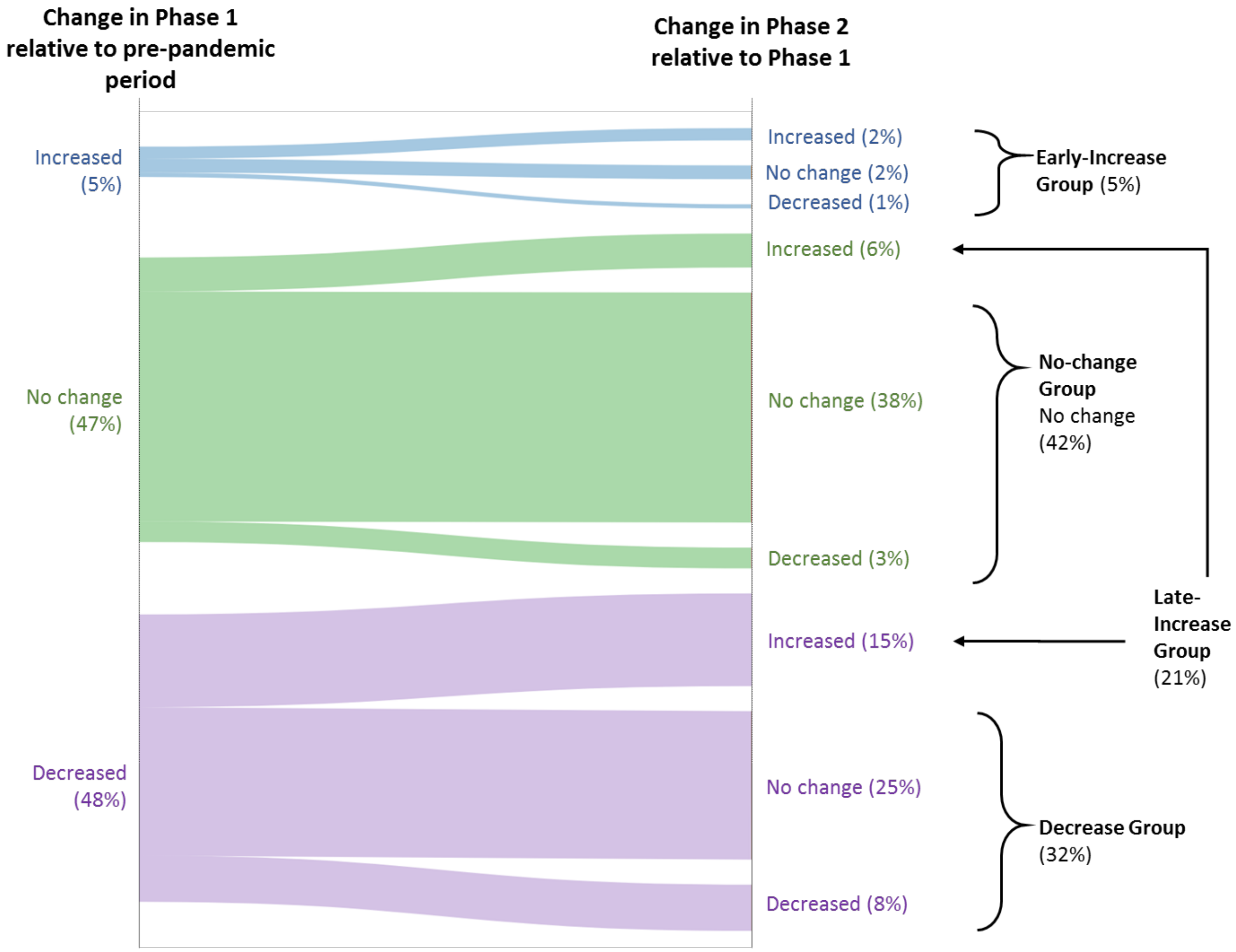

Notes: Based on 1136 responses (62 not applicable or missing excluded). Partner change groups were defined by three authors (MG, AA, HC) according to reported relative changes in partner number in phase 1 and 2 (left and right axes in above figure) based on study hypotheses and dominant behavior patterns. Assigned labels for each partner change group used in this manuscript are listed on the right hand side of the figure.

Mark Gilbert, et al. Changes in partner number and use of COVID-19 risk reduction strategies during initial phases of the pandemic in British Columbia, Canada: a survey of sexual health service clients. *Canadian Journal of Public Health.* (Corresponding author: Mark Gilbert, BC Centre for Disease Control. Mark.gilbert@bccdc.ca.)

Table: Description of Public health measures in BC

During phase 1: Enacting of public health measures (March 14-May 19, 2020)

- March 16: mass gatherings public health order implemented (>50 people)
- March 16: entry of foreign nationals banned
- March 16: symptomatic individuals banned from flights to Canada, international flights restricted to four national airports
- March 17: BC public health emergency declared
- March 17: traveller self-isolation public health order implemented
- March 18: provincial state of emergency declared
- March 18: food and drink service restrictions public health order implemented
- March 20: US/Canada border closed to non-essential travel
- March 21: closure of personal service establishments

During phase 2: Gradual lifting of public health measures (May 20, 2020 to time of survey)

- May 19: Restoration of health services, retail, hair salons, in-person counselling, restaurants, cafes, pubs, museums, libraries, office-based worksites, sports, outdoor spaces, and child care.
- June 1: Students in K-12 return to school on a gradual and part-time basis.
- June 24: Non-essential travel within BC permitted with reopening of accommodation industry (hotels, motels, RV parks, cabins, etc.), overnight camping in BC parks, motion pictures and television production, and movie theatres.

Source:

British Columbia Weekly COVID-19 surveillance report Sept 25- Oct 1, 2020, BC Centre for Disease Control. http://www.bccdc.ca/Health-Info-Site/Documents/BC_Surveillance_Summary_Oct_01_2020_final.pdf.

Survey: Sex in the Time of COVID-19 Survey Instrument

PART 1 of 3: Sex in the time of COVID-19

Thank you for participating! We'd like to start by asking whether COVID-19 has had an impact on your sex life.

1. **At the beginning of the COVID-19 pandemic (March 2020), were you…?**
   (1) Single (2) In a monogamous relationship with one person (3) In an open relationship with one person (4) In a monogamous relationship with more than one person (5) In an open relationship with more than one person (6) Other type of relationship (7) Prefer not to answer

2. **In the year prior to the COVID-19 pandemic, who did you have sex with? (Check all that apply)**
   (1) Men (inclusive of trans men) (2) Women (inclusive of trans women) (3) Non-binary people (4) No one

3. **Since the beginning of the COVID-19 pandemic in BC (March 2020), how many sex partners have you had?**
   (1) 0 (2) 1 (3) 2-3 (4) 4-5 (5) 6-9 (6) 10 or more (7) Prefer not to say

   3a. If any: **How would you describe these sex partners?** (Check all that apply)
   (1) Person(s) I have sex with regularly (2) New sexual partner (3) Casual partner/hookup (4) Group sex (5) None of the above (6) Prefer not to say

   3b.i. If 1 partner: **Do you live with this partner?**
   (1) Yes (2) No (3) Prefer not to say

   3b.ii If any, and more than 1 partner: **Do you live with any of these sex partners?**
   (1) Yes, all of them (2) Yes, some of them (3) No, none of them (4) Prefer not to say

4. **This next question asks you about the first phase or first few months after the COVID-19 pandemic began in BC (March to mid-May 2020). Did any of the following change for you, compared to before the pandemic began?**

	Decreased	No change	Increased	Not applicable
Interest in sex	( )	( )	( )	( )
Use of pornography	( )	( )	( )	( )
Number of sexual partners	( )	( )	( )	( )
Opportunities to have sex	( )	( )	( )	( )
Use of dating/hook-up apps to connect online with other people	( )	( )	( )	( )
Your use of dating/hook-up apps to meet other people in person	( )	( )	( )	( )

Use of condoms	( )	( )	( )	( )
Use of marijuana	( )	( )	( )	( )
Taking PrEP	( )	( )	( )	( )
Use of other recreational drugs	( )	( )	( )	( )
Use of alcohol	( )	( )	( )	( )
Sex work	( )	( )	( )	( )

5. **Now we'd like you to think about the months after the first phase of the COVID-19 pandemic, when services began opening again (mid-May to present). Compared to the first phase (March to mid-May) of the COVID-19 pandemic have any of the following changed for you?**

	Decreased	No change	Increased	Not applicable
Interest in sex	( )	( )	( )	( )
Use of pornography	( )	( )	( )	( )
Number of sexual partners	( )	( )	( )	( )
Opportunities to have sex	( )	( )	( )	( )
Use of dating/hook-up apps to connect online with other people	( )	( )	( )	( )
Use of dating/hook-up apps to meet other people in person	( )	( )	( )	( )
Use of condoms	( )	( )	( )	( )
Taking PrEP	( )	( )	( )	( )
Use of marijuana	( )	( )	( )	( )
Use of other recreational drugs	( )	( )	( )	( )
Use of alcohol	( )	( )	( )	( )
Sex work	( )	( )	( )	( )

6. **In the first few months after the pandemic began (March to mid-May 2020), how worried were you about getting COVID-19?**
(1) Not at all worried (2) Somewhat worried (3) Very worried (4) Extremely worried (5) Prefer not to say

(Corresponding author: Mark Gilbert, BC Centre for Disease Control. Mark.gilbert@bccdc.ca.)

7. **Today, how has your worry about getting COVID-19 changed, compared to the months after the pandemic began (March to mid-May 2020)?**
   (1) I am more worried (2) My level of worry hasn't changed (3) I am less worried (4) Prefer not to say

8. **In the first few months after the pandemic began (March to mid-May 2020), how worried were you about the chance of being exposed to COVID-19 during sexual encounters?**
   (1) Not at all worried (2) Somewhat worried (3) Very worried (4) Extremely worried (5) Prefer not to say

9. **Today, how has your worry about being exposed to COVID-19 during sexual encounters changed, compared to the months after the pandemic began (March to mid-May 2020)?** (1) I am more worried (2) My level of worry hasn't changed (3) I am less worried (4) Prefer not to say

10. **Have you looked for, or received, information from any of the following sources about the risk of being exposed to COVID-19 infection during sexual encounters?** (check all that apply)
    (1) By searching online (2) Through social media (Facebook, Twitter, etc) (3) Through news media (TV, radio, newspapers) (4) From a public health agency website (e.g., the BC Centre for Disease Control website) (5) From a community-based organization (6) From friends or family (7) From a relationship/sexual partner (8) From a healthcare provider (9) Other, please specify: _____ (10) Have not looked for or received information about COVID-19 and sex

11. **There are a number of strategies that people may be using to reduce their risk of getting COVID-19 infection during sexual encounters or passing to their partner(s). Since the beginning of the pandemic, have you done any of the following to reduce your risk?** (Check all that apply)

    - Not having sex
    - More masturbating / sex with yourself
    - Limiting sex to a person/people you live with
    - Limiting sex to a small number of regular partners ("a bubble")
    - Having online or virtual sex
    - Reducing your number of casual sex partners
    - Asking your sex partner if they are experiencing COVID-19 symptoms
    - Asking your sex partner about the precautions they are taking to reduce their risk of COVID-19
    - Avoiding having sex if you're feeling unwell or have symptoms of COVID-19
    - Avoiding kissing or saliva exchange/contact
    - Avoiding rimming
    - Wearing a face mask during sex
    - Avoiding sexual positions with close face to face contact
    - Avoiding group sex
    - Washing your hands with soap and water, before and after sex
    - Washing any shared sex toys with soap and water, before and after sex
    - Other, please describe:_______
    - None of the above

PART 2 of 3: Need for sexual health services during COVID-19

The next section of the survey shifts to questions about your use of sexual health services before and after the start of the COVID-19 pandemic in BC (March 2020.

12. **How often did you usually get tested for sexually transmitted infections (STIs), before the beginning of the pandemic?**
    (1) Have only tested once (2) Every few years (3) Once a year (4) Twice a year (5) A few times per year (e.g. every 3-4 months) (6) Once a month (7) No set pattern (8) Prefer not to say

13. **In the year prior to the COVID-19 pandemic, did you get tested for STIs through any of the following services?** (Check all that apply)
    (1) BCCDC Clinic at 655 West 12th Ave (2) Bute Street clinic at Qmunity (3) GetCheckedOnline (4) Health Initiative for Men clinic (5) Options for Sexual Health Clinic (6) Island Sexual Health Clinic (7) Other clinic, please describe: ____ (8) Did not need to get tested (9) Prefer not to say

14. **Since the beginning of the COVID-19 pandemic, have you wanted or needed to get tested for STIs?**
    (1) Yes, for a new, specific reason (e.g., symptoms, after a specific event, new partner, partner with STI)
    (2) Yes, according to my usual testing routine (e.g., due for an STI test)
    (3) No

    14a. (If 1, 2 to Q14) **Did you get tested for STIs at this time?**
    (1) Yes (2) No; *skip to Q15*

    14b. (If 1 to Q14a.) **Where did you get tested?**
    (1) BCCDC Clinic at 655 West 12th Ave (2) Through GetCheckedOnline (3) Health Initiative for Men clinic (4) Options for Sexual Health Clinic (5) Island Sexual Health Clinic (6) Family doctor or nurse practitioner (7) Walk-in clinic (8) Other, please describe: _______________

15. **Since the beginning of the COVID-19 pandemic, have you wanted or needed to access sexual health services for any reason besides STI testing?** (Check all that apply)
    (1) Speak with a health care provider about a sexual health concern (e.g., symptoms, questions)
    (2) Speak with a health care provider about a mental health concern
    (3) Access birth control
    (4) Get a pregnancy test
    (5) Access treatment for a new STI (e.g., syphilis, chlamydia, gonorrhea)
    (6) Access to treatment for on-going symptoms (e.g., warts, herpes)
    (7) Access to Pre-exposure prophylaxis (PrEP)
    (8) Access to condoms
    (9) Access to harm reduction supplies
    (10) Other, describe: ________________
    (11) No need to access sexual health services

    15a. (If 1-10 to Q15) **Did you get these sexual health services you needed at this time?**
    (1) Yes (2) No; *skip to Q16*

    15b. (If 1 to 15a) **Where did you get this sexual health service?**
    (1) BCCDC Clinic at 655 West 12th Ave (2) Health Initiative for Men clinic (3) Options for Sexual Health Clinic (4) Island Sexual Health Clinic (5) Family doctor or nurse practitioner (6) Walk-in clinic (7) Other, please describe: _______________

16. (If wanted or needed to access testing or sexual health services in Q14 and/or Q15) **Did any of the following factors lead you to <u>avoid or delay</u> seeking testing or sexual health care during the COVID-19 pandemic?** (Check all that apply)
    - I didn't know where to access sexual health or STI testing services
    - I was concerned about getting COVID-19 while travelling to a clinic or lab
    - I was concerned about getting COVID-19 while at a clinic or lab
    - I was worried that a healthcare provider might judge me for having sex during COVID
    - I live or am in close contact with someone at risk of COVID-19 (e.g., senior, immunocompromised)
    - The place I usually go to for testing/care was closed or had reduced services because of COVID-19
    - There was public messaging that I was not supposed to seek healthcare that wasn't urgent
    - Other reason, please describe:_____
    - I didn't avoid or delay seeking testing or sexual health care
    - Prefer not to say

17. (If in Q13 did not test through GCO) **GetCheckedOnline is a free online testing service for HIV and other sexually transmitted infections (e.g., syphilis, chlamydia, gonorrhea, hepatitis C) in BC created by the BC Centre for Disease Control.**

    GetCheckedOnline lets you skip a visit to a clinic by getting tested by printing a lab form from a website or downloading an electronic version on your phone, that you then take to a lab, and then get your results online or by phone.

    Before today, did you know about GetCheckedOnline?
    (1) Yes (2) No (3) Not sure

    17a. (If Yes to Q17) **Have you ever been tested through GetCheckedOnline?**
    (1) Yes (2) No (3) Not sure

18. **How much do you agree or disagree with the statements below, based on how you are feeling right now?**
    - I am able to get the sexual health care I need during the COVID-19 pandemic
    - I am comfortable accessing in-person sexual health and testing services during the COVID-19 pandemic
    - I would feel ashamed if people knew about my sex life during the COVID-19 pandemic
    - My satisfaction with my sex life has not changed during the COVID-19 pandemic
    - Other people will judge me for having sex during the COVID-19 pandemic
    - I prefer to get tested through GetCheckedOnline because of the COVID-19 pandemic
    - I am, or I will soon be, having sex with more people than I was earlier in the COVID-19 pandemic

    R: (for each item): (1) Strongly agree (2) Agree (3) Neither agree nor disagree (4) Disagree (5) Strongly disagree

19. **How likely or unlikely would you be to use the following sexual health services, if available?**

    - **Video visit with a sexual health care provider to discuss your sexual health**
    - **Phone call with a sexual health care provider to discuss your sexual health**

- **Texting with a sexual health care provider to discuss your sexual health**
- **Text messaging service that provides STI test results**
- **Text messaging service that provides reminders (e.g., to take medication, for appointments)**
- **Receiving test kits or antibiotics at home by mail (in plain packaging)**
- **Sending a picture of a rash or lesion to a sexual health care provider**

(1) Very likely (2) Likely (3) Neither Likely nor unlikely (4) Unlikely (5) Very unlikely

20. **Suppose you could get tested through a self-collection kit, where you could collect your own specimens at home and return them to a clinic or lab for testing. How likely or unlikely would you be to use this service?**
(1) Very likely (2) Likely (3) Neither likely nor unlikely (4) Unlikely (5) Very unlikely

20a. (If 1 or 2 to Q20) **What samples could you self-collect, if detailed instructions were provided?** (Check all that apply)
(1) Prick your finger to provide a few drops of blood (2) Pee into a container (urine) (3) swab your throat (4) swab your bum (rectum) (5) swab your vagina /front hole (6) I would not self-collect any of the above

20b. How would you most prefer to receive the self-collection kit?
(1) By mail (2) By picking the kit up at a clinic (3) By picking the kit up at a lab (4) no preference

21. **Suppose there was an "express testing" service, where after an initial assessment by phone or video, you could go to a clinic to have specimens collected by a health care provider. How likely or unlikely would you be to use this service?**
(1) Very likely (2) Likely (3) Neither Likely nor unlikely (4) Unlikely (5) Very unlikely

Part 3 of 3: About you

This next section will ask some questions about you. The information will help us to learn more about the people who have used the sexual health services offered by the BC Centre for Disease Control and help us to see how different groups of people may have been affected by the COVID-19 pandemic.

22. **What are the first three characters of your postal code?**
[open text box] ______

23. **How old are you?**
_____ years old

24. **What is your gender identity?**
(1) Man (2) Woman (3) Non-binary (4) Genderfluid (5) Other, please specify: _______ (6) Prefer not to say

25. **Do you identify as transgender, have lived experience as trans, or have a history of gender transition?**
(1) Yes (2) No (3) Prefer not to say

26. **What best describes your sexual identity?**
(1) Straight (heterosexual) (2) Gay/lesbian (homosexual) (3) Bisexual (4) Queer (5) Pansexual (6) Other, specify ___ (7) Prefer not to say

27. **Which of these do you identify with?** (Check all that apply)
(1) Arab, West Asian (e.g. Iranian, Afghan) (2) Black (e.g., African, Afro-Caribbean, African Canadian) (3) East Asian (e.g. Chinese, Japanese, Korean) (4) Indigenous (First Nations, Inuit, Métis) (5) Latin American (6) South Asian (e.g. East Indian, Pakistani, Sri Lankan) (7) Southeast Asian (Filipino, Vietnamese, Thai) (8) White (9) Other, please describe: ______ (11) Prefer not to say

27a. If you identify as Indigenous, do you identify as Two-Spirit?
(1) Yes (2) No (3) Prefer not to say

28. **What is the highest level of education that you have completed?**
(1) None, elementary or some high school (2) High school or equivalent (3) Post-secondary school (e.g., certificate, diploma) (4) Bachelor's degree (5) Graduate degree (Master's, PhD, MD, etc.) (6) Prefer not to say

29. **What was your employment status before the beginning of the COVID-19 pandemic?** (Check all that apply)
(1) Employed full-time (30+ hours/week) (2) Employed part-time (<30 hours/week) (3) Self-employed (e.g., professional, contractor, business owner) (4) On government assistance (e.g., E.I.) (5) On disability (e.g., long term disability, disability pension, PWD) (6) Student (7) Retired (8) Unemployed (9) Unable to work (10) Prefer not to say

30. **What was your income (before tax) in 2019?**
(1) <$20,000 (2) $20,000-$39,999 (3) $40,000-$59,999 (4) $60,000-$79,999 (5) $80,000 or more (6) Prefer not to say

31. **Since the beginning of the COVID-19 pandemic (March 2020), how has it been for you or your household to meet its financial needs?**
(1) Much more difficult (2) Somewhat more difficult (3) Neither more difficult nor easier (4) Somewhat easier (5 Much easier (6) I don’t know (7) Prefer not to answer

32. **In the first few months after the pandemic began (March to mid-May 2020), how would you have rated your mental health?**
(1) Excellent (2) Very good (3) Good (4) Fair (5) Poor

33. **Today, compared to the months after the pandemic began (March to mid-May 2020), would you say that your mental health has:**
(1) Worsened by a lot (2) Worsened by a little (3) Stayed about the same (4) Improved by a little (5) Improved by a lot (6) Prefer not to say

END SURVEY PAGE

Mark Gilbert, et al. Changes in partner number and use of COVID-19 risk reduction strategies during initial phases of the pandemic in British Columbia, Canada: a survey of sexual health service clients. *Canadian Journal of Public Health.* (Corresponding author: Mark Gilbert, BC Centre for Disease Control. Mark.gilbert@bccdc.ca.)

Checklist for Reporting Results of Internet E-Surveys (CHERRIES)

Item Category	***Checklist Item***	***Explanation***	***From manuscript***
**Design**			
	Describe survey design	Describe target population, sample frame. Is the sample a convenience sample? (In "open" surveys this is most likely.)	Lines 162-163. The study is described as a cross-section email survey.
**IRB (Institutional Review Board) approval and informed consent process**			
	IRB approval	Mention whether the study has been approved by an IRB.	Lines 230-232. Ethics approval was granted by UBC's behavioral research ethics board.
	Informed consent	Describe the informed consent process. Where were the participants told the length of time of the survey, which data were stored and where and for how long, who the investigator was, and the purpose of the study?	Lines 200-202. The Survey landing page provided necessary information of informed consent.<br><br>The landing page also indicated the survey was expected to take 15 minutes, the name of the investigator, and the purpose of the survey.
	Data protection	If any personal information was collected or stored, describe what mechanisms were used to protect unauthorized access.	Not applicable – personal identifiers not collected in the survey.
**Development and pre-testing**			
	Development and testing	State how the survey was developed, including whether the usability and technical functionality of the electronic questionnaire had been tested before fielding the questionnaire.	Lines 175-177. Survey items were adapted from literature/prior research, or developed with input from sexual health service providers and a Community Advisory Board. The survey was pilot tested with eight members of the CAB and revised accordingly.
**Recruitment process and description of the sample having access to the questionnaire**			
	Open survey versus closed survey	An "open survey" is a survey open for each visitor of a site, while a closed survey is only open to a sample which the investigator knows (password-protected survey).	Not applicable. E-mail survey to existing clients consenting to be contacted for research purposes.

Item Category	***Checklist Item***	***Explanation***	***From manuscript***
	Contact mode	Indicate whether or not the initial contact with the potential participants was made on the Internet. (Investigators may also send out questionnaires by mail and allow for Web-based data entry.)	Lines 195-199.
	Advertising the survey	How/where was the survey announced or advertised? Some examples are offline media (newspapers), or online (mailing lists – If yes, which ones?) or banner ads (Where were these banner ads posted and what did they look like?). It is important to know the wording of the announcement as it will heavily influence who chooses to participate. Ideally the survey announcement should be published as an appendix.	Not applicable.
**Survey administration**			
	Web/E-mail	State the type of e-survey (eg, one posted on a Web site, or one sent out through e-mail). If it is an e-mail survey, were the responses entered manually into a database, or was there an automatic method for capturing responses?	Lines 198-216. The survey was distributed by e-mail, with link to an online survey using REDCap. Data was downloaded and stored in a secure data repository.
	Context	Describe the Web site (for mailing list/newsgroup) in which the survey was posted. What is the Web site about, who is visiting it, what are visitors normally looking for? Discuss to what degree the content of the Web site could pre-select the sample or influence the results. For example, a survey about vaccination on a anti-immunization Web site will have different results from a Web survey conducted on a government Web site	Not applicable.
	Mandatory/voluntary	Was it a mandatory survey to be filled in by every visitor	Line 200-201. Survey was voluntary.

Item Category	***Checklist Item***	***Explanation***	***From manuscript***
		who wanted to enter the Web site, or was it a voluntary survey?	
	Incentives	Were any incentives offered (eg, monetary, prizes, or non-monetary incentives such as an offer to provide the survey results)?	Lines 208 – 209. Draw for a $200 gift card.
	Time/Date	In what timeframe were the data collected?	Line 163. July 21 & Aug 4, 2020.
	Randomization of items or questionnaires	To prevent biases items can be randomized or alternated.	Survey items were not randomized.
	Adaptive questioning	Use adaptive questioning (certain items, or only conditionally displayed based on responses to other items) to reduce number and complexity of the questions.	Line 179. Adaptive questioning was used.
	Number of Items	What was the number of questionnaire items per page? The number of items is an important factor for the completion rate.	Line 178. 33 items
	Number of screens (pages)	Over how many pages was the questionnaire distributed? The number of items is an important factor for the completion rate.	Line 178. One item per page.
	Completeness check	It is technically possible to do consistency or completeness checks before the questionnaire is submitted. Was this done, and if "yes", how (usually JAVAScript)? An alternative is to check for completeness after the questionnaire has been submitted (and highlight mandatory items). If this has been done, it should be reported. All items should provide a non-response option such as "not applicable" or "rather not say", and selection of one response option should be enforced.	There were no in-survey completeness or consistency checks. No survey items were mandatory. With most survey items, an option such as "prefer not to say" was included.
	Review step	State whether respondents were	Lines 179-181. Participants

Item Category	*Checklist Item*	*Explanation*	*From manuscript*
		able to review and change their answers (eg, through a Back button or a Review step which displays a summary of the responses and asks the respondents if they are correct).	could go backwards and forwards in the survey to review or edit answers, and could save answers and continue the survey at a later date.
**Response rates**			
	Unique site visitor	If you provide view rates or participation rates, you need to define how you determined a unique visitor. There are different techniques available, based on IP addresses or cookies or both.	Not applicable for an e-mail survey.
	View rate (Ratio of unique survey visitors/unique site visitors)	Requires counting unique visitors to the first page of the survey, divided by the number of unique site visitors (not page views!). It is not unusual to have view rates of less than 0.1 % if the survey is voluntary.	Not applicable for an e-mail survey.
	Participation rate (Ratio of unique visitors who agreed to participate/unique first survey page visitors)	Count the unique number of people who filled in the first survey page (or agreed to participate, for example by checking a checkbox), divided by visitors who visit the first page of the survey (or the informed consents page, if present). This can also be called "recruitment" rate.	Lines 244-246 describe the participation rate (percent who started the survey out of all clients invited to the survey)
	Completion rate (Ratio of users who finished the survey/users who agreed to participate)	The number of people submitting the last questionnaire page, divided by the number of people who agreed to participate (or submitted the first survey page). This is only relevant if there is a separate "informed consent" page or if the survey goes over several pages. This is a measure for attrition. Note that "completion" can involve leaving questionnaire items blank. This is not a measure for how completely questionnaires were filled in. (If you need a measure for this, use the word "completeness rate".)	Lines 214-215. Survey completion tracked by clicking on submit at end of survey.<br><br>Lines 244-246 describe the completion rate.

Item Category	***Checklist Item***	***Explanation***	***From manuscript***
**Preventing multiple entries from the same individual**			
	Cookies used	Indicate whether cookies were used to assign a unique user identifier to each client computer. If so, mention the page on which the cookie was set and read, and how long the cookie was valid. Were duplicate entries avoided by preventing users access to the survey twice; or were duplicate database entries having the same user ID eliminated before analysis? In the latter case, which entries were kept for analysis (eg, the first entry or the most recent)?	This was an e-mail survey using RedCAP, which does not use cookies or prevent multiple entries.
	IP check	Indicate whether the IP address of the client computer was used to identify potential duplicate entries from the same user. If so, mention the period of time for which no two entries from the same IP address were allowed (eg, 24 hours). Were duplicate entries avoided by preventing users with the same IP address access to the survey twice; or were duplicate database entries having the same IP address within a given period of time eliminated before analysis? If the latter, which entries were kept for analysis (eg, the first entry or the most recent)?	This was an e-mail survey using RedCAP, which does not track IP addresses.
	Log file analysis	Indicate whether other techniques to analyze the log file for identification of multiple entries were used. If so, please describe.	No other methods used.
	Registration	In “closed” (non-open) surveys, users need to login first and it is easier to prevent duplicate entries from the same user. Describe how this was done. For example, was the survey never displayed a second time once the user had filled it in, or was the username	Not applicable.

Item Category	*Checklist Item*	*Explanation*	*From manuscript*
		stored together with the survey results and later eliminated? If the latter, which entries were kept for analysis (eg, the first entry or the most recent)?	
**Analysis**			
	Handling of incomplete questionnaires	Were only completed questionnaires analyzed? Were questionnaires which terminated early (where, for example, users did not go through all questionnaire pages) also analyzed?	Line 219. Only completed (submitted) questionnaires were analyzed.<br><br>Of the 230 surveys which were not completed (submitted), most attrition occurred prior to the questions of interest for this study (i.e., 63% dropped off prior to questions regarding partner number).
	Questionnaires submitted with an atypical timestamp	Some investigators may measure the time people needed to fill in a questionnaire and exclude questionnaires that were submitted too soon. Specify the timeframe that was used as a cut-off point, and describe how this point was determined.	Not applicable.
	Statistical correction	Indicate whether any methods such as weighting of items or propensity scores have been used to adjust for the non-representative sample; if so, please describe the methods.	Not applicable.
